# Optically gated beating-heart imaging

**DOI:** 10.3389/fphys.2014.00481

**Published:** 2014-12-11

**Authors:** Jonathan M. Taylor

**Affiliations:** School of Physics and Astronomy, University of GlasgowGlasgow, UK

**Keywords:** optical gating, heart imaging, cardiac imaging, heart synchronization, motion compensation, 3D imaging

## Abstract

The constant motion of the beating heart presents an obstacle to clear optical imaging, especially 3D imaging, in small animals where direct optical imaging would otherwise be possible. Gating techniques exploit the periodic motion of the heart to computationally “freeze” this movement and overcome motion artifacts. Optically gated imaging represents a recent development of this, where image analysis is used to synchronize acquisition with the heartbeat in a completely non-invasive manner. This article will explain the concept of optical gating, discuss a range of different implementation strategies and their strengths and weaknesses. Finally we will illustrate the usefulness of the technique by discussing applications where optical gating has facilitated novel biological findings by allowing 3D *in vivo* imaging of cardiac myocytes in their natural environment of the beating heart.

## 1. Introduction

Optical imaging of the heart in its natural state is essential to cellular-level studies of early heart development, for which animal models such as the mouse, chick, and zebrafish are widely used due to the optical accessibility of the heart. The constant motion of the beating heart presents significant challenges, particularly for 3D imaging, but the periodic nature of this motion can be exploited through the use of gated (or synchronized) imaging. Optical gating is an increasingly popular method for “freezing” the motion of the heart in an entirely non-invasive manner, normally using the image data itself to determine the *phase* of the heart on each acquisition (in other words the time point within the heartbeat, such as end-systole).

In order to build up a three-dimensional (3D) image of an object, it is generally necessary to acquire a series of sequential 2D images. For example, in the case of a spinning-disk confocal microscope, individual image “slices” in the *xy* plane can be acquired relatively quickly, but the focus must be scanned successively through the sample in order to acquire a “*z* stack” of 2D images from which the 3D object can subsequently be reconstructed. In fixed tissue this sequential acquisition does not present problems, but in living tissue motion artifacts can degrade the dataset or render it meaningless. The most extreme example of this is within the living, beating heart itself. In small vertebrates the heart may beat at rates between 2 and 10 Hz, and 3D images will be compromised by severe motion artifacts if the motion of the heart is not taken into account. Although recently-developed approaches potentially allow a full 3D image stack of the heart to be acquired fast enough to avoid substantial motion artifacts (Fahrbach et al., 2013; Mickoleit et al., 2014), this necessarily limits the number of slices that can be acquired, and requires extremely short exposure times (with implications for signal-to-noise ratio).

**Figure 1:**
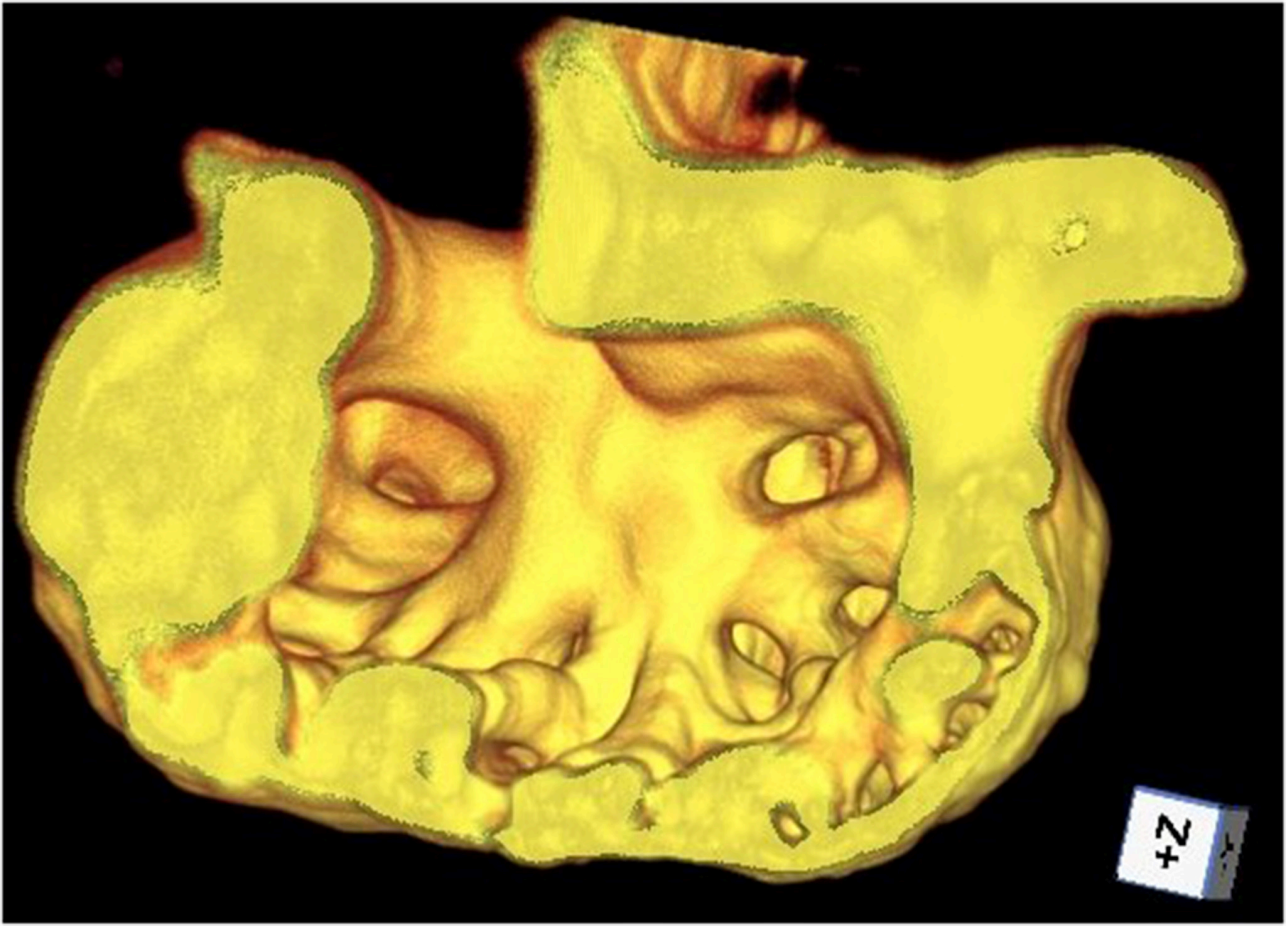
**Cutaway of trabeculated ventricle of a zebrafish heart, rendered from beating-heart data synchronized using prospective optical gating (still from video at http://www.opticsinfobase.org/boe/viewmedia.cfm?uri=boe-3-12-3043&seq=3)**. Image reproduced from Taylor et al. (2012) with permission.

However, the cyclical motion of the heart with every successive beat means that it is not necessary to image every slice simultaneously at the same instant in time. It is sufficient that each slice is acquired when the heart is in the same position: this need not necessarily be the same actual heartbeat. By acquiring each image at the same phase of the cardiac cycle, it is possible to computationally or optically “freeze” the motion of the heart and obtain a 3D image representing a snapshot in time of the heart (Figure 1).

This review will explain the principles of optical gating and the advantages that it offers. We will compare and contrast two different categories of optical gating: retrospective and prospective gating. In retrospective gating, a sequence of hundreds of images is acquired using a free-running camera and post-acquisition analysis is used to reorder the sequence into a single high-temporal-resolution representation of a single heartbeat. In prospective gating, individual images are triggered in real time at known points in the heartbeat. We will discuss the situations in which each approach is most useful, along with factors affecting the accuracy of each technique. We will show that optical gating has already been a key tool in a number of biological discoveries, and holds great potential for future impact on developmental biology.

## 2. Motivation for optical gating

Without a solution to the problem of heart motion, it may be necessary to stop the heart completely, at least for the duration of imaging. In the mouse, Butcher et al. (2007) perfused the heart with a polymer which subsequently solidified in order to take a “cast” of the heart that could be imaged without motion artifacts. Panáková et al. (2010) used the drug blebbistatin to pharmacologically uncouple the heart muscle in zebrafish in order to observe the electrical activity of the heart in the absence of motion, and while (Staudt et al., 2014) performed imaging using retrospective gating they also pharmacologically stopped the heart using a high dose of the anesthetic Tricaine (MS-222) in order to perform high resolution cell-level imaging. In addition to the drastic impact this has on normal physiological function, a stopped heart will lose its tone due to lack of blood pressure and resistance to pumping, and it will take on a shape that does not represent its normal morphology at *any* phase in the healthy heartbeat (Mickoleit et al., 2014)—thus limiting the relevance of the observations.

As a preface to our discussion of optical gating we will briefly survey various non-optical techniques for beating-heart imaging. A widely-used gating method is ECG-gating, where the electrocardiogram is monitored in real time and e.g., the R wave is used to trigger image acquisition. This is widely used for human MRI (Jerecic et al., 2004). However, in MRI there is the specific difficulty of interaction between the radio-frequency fields used for imaging and the electrical probes used to monitor the ECG. Thus, retrospective self-gating based on the MR signal itself is commonly used in humans (Crowe et al., 2004; Buehrer et al., 2008) and in mice (Bishop et al., 2006; Heijman et al., 2007), where the difficulties of ECG measurement during MRI are particularly pronounced.

In mice ECG gating is routinely used for optical imaging too (see Bartling et al., 2010; Vinegoni et al., 2014 for reviews). Additionally, physical immobilization may be used to reduce the level of motion in the exposed but intact heart (Lee et al., 2012; Vinegoni et al., 2014). However, ECG measurement is exceptionally challenging in smaller animals such as the zebrafish (Dhillon et al., 2013) and chick (Jenkins et al., 2007) where it is generally too cumbersome for routine imaging, although wearable microelectrode arrays have recently been reported for monitoring of cardiac electrical activity in adult zebrafish and neonatal mice (Cao et al., 2014).

A more drastic intervention that can be used is “pacing” of the heart. In the case of the chick and mouse, carefully-placed electrodes can generate a voltage pulse to trigger heart contraction (Jenkins et al., 2006) in an approach analogous to the use of pacemaker devices in human hearts. Image acquisition can be triggered using the same equipment that is used to stimulate the heart, thereby ensuring that all images are taken at the same point in the cycle. However, in the case of small animals the physical damage due to electrode placement cannot be ignored. A further disadvantage is that stable pacing can normally only be achieved at heart rates greater than the intrinsic pacing frequency of the heart, unless the sino-atrial node is ablated.

A more recent alternative that has emerged is optical pacing. Here light itself is used to influence and regularize the heart rate. This could be viewed as a form of optically-triggered prospective gating, albeit one that involves a non-contact intervention to manipulate and pace the natural rhythm of the heart. Jenkins et al. (2010) used pulsed infrared laser light with a threshold power of around 0.8 J/cm^2^ and were able to optically pace the embryonic quail heart rate at between 2 and 3 Hz. The embryo had not been subject to any genetic manipulation, and the pacing resulted from a direct response of the wild-type cells to the high intensity laser light incident on them, with various possible mechanisms suggested by the authors. Transmission electron micrographs revealed no obvious damage to the cardiomyocytes at powers close to threshold, although at higher powers (4.3 J/cm^2^ per pulse) substantial tissue damage was apparent. Arrenberg et al. (2010) used a genetically modified zebrafish line in which light-sensitive channelrhodopsin was expressed in cardiomyocytes, and optically stimulated the cells using light from a lamp that was switched and masked using a digital micromirror device. This enabled them to pace the heart at rates between 2.7 and 4.7 Hz using energy densities two orders of magnitude lower, at 2 mJ/cm^2^ per pulse (private communication), although we note that the laser spot size was reported to be somewhat broader in the former case.

Electrical or optical pacing of the heart allows the periodicity of the heart to be precisely controlled, allowing for high temporal resolution prospective gating of images. However, depending on the nature of the study it may be necessary to consider whether the electrical activity of the heart has been substantially altered by this intervention, or even whether the precise form of the contraction itself may have been altered.

## 3. Optical gating methods

Optical gating represents the least invasive approach for dealing with tissue motion in the heart, in the sense that no additional interaction with the specimen is required beyond that necessary for imaging. Furthermore, the fact that the heart continues to beat normally, and without pacing, means that the observations represent the natural physical and electrophysiological state of the heart. In practice two main factors will determine the quality of the “snapshot” that can be obtained of the heart, and ultimately determine the effective resolution of the resultant 3D image.

Firstly, the integration time of an individual acquisition will induce motion blur. In a static image, the resolution is determined by the point spread function (PSF), the size over which a point feature in the image will be blurred in the image. Even in a single 2D image, if over the integration time of the image a feature has moved by a distance greater than the size of the PSF then the effective PSF will be broadened (Vermot et al., 2008)—in other words the image will be further blurred due to the motion. Mathematically, the PSF will be convolved with the trajectory of the moving feature over the course of the integration time.

Secondly, images must be acquired at the correct time to match the desired phase of the heart, or otherwise spatial errors will be present in the image. If a feature is moving with an instantaneous velocity **v** then a timing error of δ*t* for the image acquisition will result in an error of **v**δ*t* in the feature's position. Spatial errors prevent correct registration between adjacent image slices in a *z* stack, or between images that should in principle be identical. For example, if a feature is moving in *z* then it might appear in multiple image slices in a *z* stack, or shifts between adjacent slices might “break up” what should be a contiguous linear structure. A reconstructed 3D image will not then represent a true snapshot of the heart at one instant in time.

Thus, in the same way as the resolution of the imaging system must be appropriate to the scale of the features that the researcher wishes to resolve, the integration time and required timing resolution of the gating are determined by the intended experiment. In the case of a relatively coarsely spaced *z* stack intended to estimate the instantaneous volume of the heart, for example, the necessary spatiotemporal registration accuracy required between slices could be one tenth of a cycle (which for the zebrafish would make it of the order of 30 ms). On the other hand sub-micron 3D resolution would require timing accuracies of substantially less than 1 ms (based on zebrafish heart wall velocities that can be as high as 1 mm/s—Vermot et al., 2008).

Important characteristics defining the performance of a gating scheme are: temporal precision, number of raw images required, computational demands, and requirements for specialized hardware or experimental preparation. In the following sections we will describe the algorithms and setups used for retrospective and prospective gating in a single image plane, discussing them in the context of these performance characteristics, and then explain how these approaches can be extended to synchronize a full 3D data stack.

### 3.1. Retrospective gating

The purpose of *retrospective gating* is to process a video sequence spanning multiple heartbeats, labeling and sorting the frames according to their phase to allow frames to be assigned to known phases in the heart cycle. This could be in order to synchronize slice sequences taken at different depths within the heart (“inter-slice registration,” discussed further in Section 3.3), or it could be to upsample a video taken at a low framerate in order to synthesize a higher-framerate depiction of a single heartbeat.

**Figure 2:**
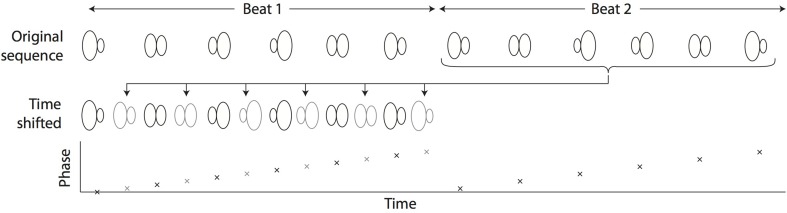
**Cartoon illustration of a video sequence consisting of images spanning two consecutive heartbeats**. By time-shifting the frames that belong to the second heartbeat, it is possible to interleave the frames and synthesize a sequence spanning a single heartbeat but at twice the framerate. The graph depicts the phase of each frame, although it is important to emphasize that post-acquisition synchronization algorithms do not generally calculate the phase directly. The correct time shifts are determined by the SLM method described in the text, and the phases are implicitly determined by the chosen time shifts.

In the case of upsampling, consider a 100 fps video sequence of a perfectly periodic heart beating at 2 Hz. Frames 10, 60, 110… (with timestamps of 0.1 s, 0.6 s, 1.1 s) would all represent exactly the same phase. However, in practice the acquisition framerate will not be a perfect multiple of the heart rate, which means that frames from subsequent beats can be used to “fill in the gaps” and obtain a synthesized higher-framerate representation of a single heartbeat by reordering the frames and interleaving images from successive heartbeats (Figure 2).

Slice synchronization, on the other hand, involves time-shifting sequences relative to each other (and possibly also warped and/or interpolated) in order to temporally align sequences representing different slices through the heart, matching phases in order to synthesize a self-consistent 3D dataset. However, in the absence of upsampling or interpolation, acquisition framerates directly determine the achievable temporal accuracy. Acquisition framerates described in the literature range from 50 fps [for OCT, Bhat et al. (2009)] to 120 fps [for confocal slit microscopy, Liebling et al. (2006a)]. We note that modern scientific CMOS cameras are capable of imaging at around 400 fps for a 512 × 512 pixel image, giving the potential for faster acquisition if the imaging modality permits.

The computationally complex aspect of retrospective gating is the post-acquisition analysis to determine the temporal shifts required to correctly align image sequences from multiple heartbeats. Two main computational techniques can be used to achieve this alignment: inter-frame correlation, and string length minimization.

When using inter-frame correlation, sequences are resampled to give an integer number of frames per heart period, and then the correct shift is identified by minimizing a similarity criterion (e.g., least-squares) between frames from the two image sequences for a given relative alignment (Liebling et al., 2005). Such a comparison can be facilitated through the use of signal processing algorithms such as the Fast Fourier Transform.

Liebling et al. (2006b) later introduced a more sophisticated synchronization approach that did not simply apply a relative time shift to align two candidate sequences. In addition to this shift, the sequences were “temporally warped” to account for variations in heart rate during acquisition of the image sequence, thus improving the temporal accuracy of the time-shifted sequence. Although this makes the synchronization problem substantially more computationally complex, they were able to render it tractable by considering a discrete subset of time shifts and then applying a dynamic programming algorithm to efficiently solve for the optimal temporal warping function.

**Figure 3:**
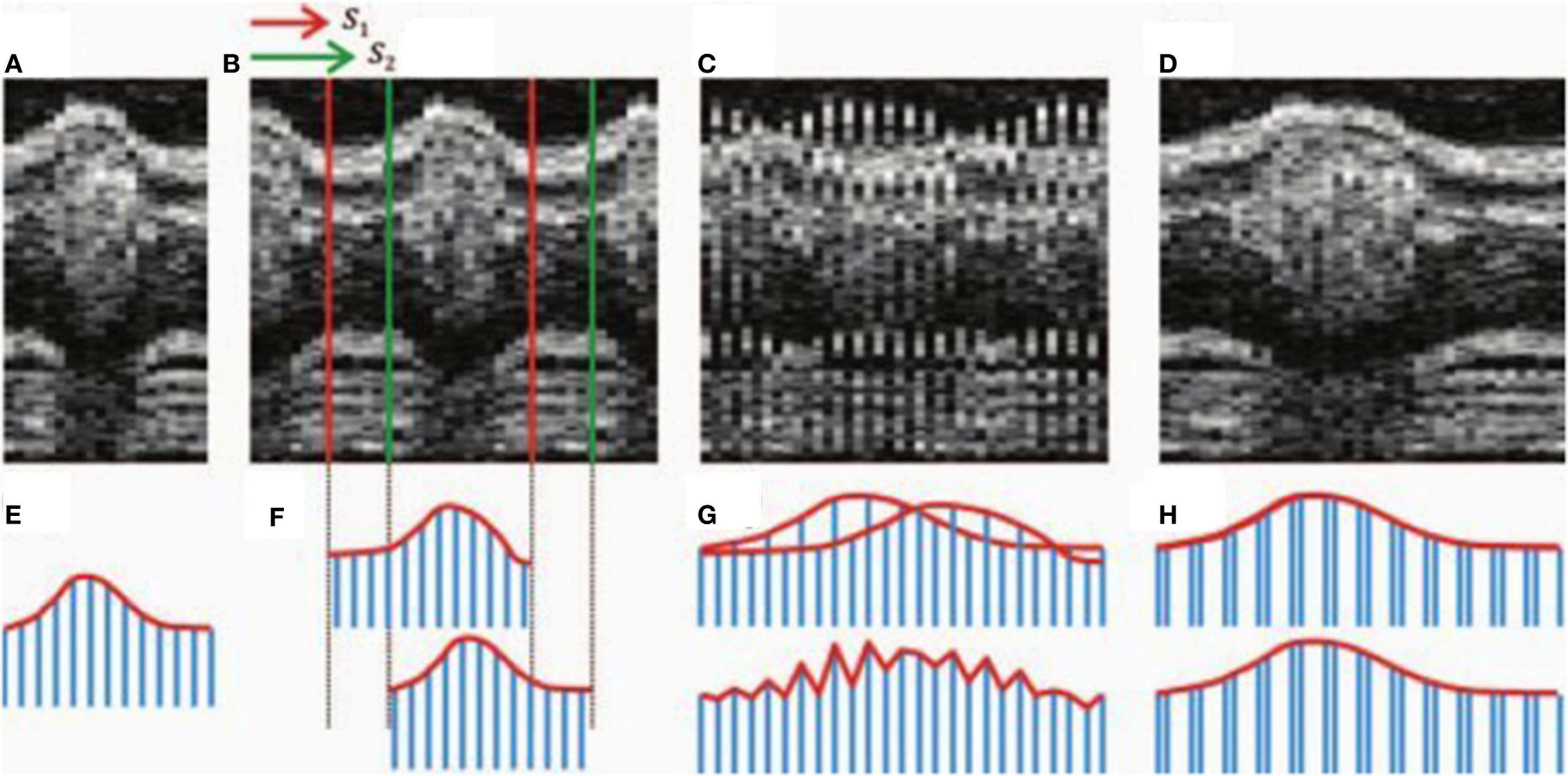
**Illustration of sequence alignment by string length minimization**. Upper row: OCT time series; lower row: string analogy. A candidate sequence (**A, E**) is compared against a second sequence (**B, F**) with two different shifts *S*_1_ and *S*_2_. If an incorrect shift is chosen (**C**) then the mismatch between adjacent slices results in a greater string length (**G**) than if the correct shift is chosen (**D, H**). Images reproduced from Happel et al. (2011) with permission.

In the case of string length minimization, two candidate image sequences are interleaved and differences are calculated between temporally adjacent frames, both in terms of their assigned times/phases and in terms of their image similarity. These differences can be interpreted as distances in a multidimensional phase space (Figure 3). The total distance will be minimized if there is a progressive change between a series of phase-adjacent frames (as would be expected from a realistic and smoothly-varying video representation of the heart), whereas the calculated length will be greater if out-of-order frames (with conflicting appearances) lie interleaved. The alignment problem is thus expressed as a minimization problem that can be solved to determine the correct ordering of the image sequence. Image similarity may be calculated in the spatial domain (Gargesha et al., 2009 used the pixelwise sum-squared-differences) or in the spectral domain (Liebling et al., 2005 compared wavelet coefficients). String length minimization has been used in conjunction with phase-wrapping in order to estimate the heart period (Liebling et al., 2005), to increase the effective framerate (Happel et al., 2011) or to synchronize sequences from two nearby depths within the heart.

An advantage common to all post-acquisition synchronization techniques is that the synchronization phase does not need to be selected in advance—it only needs to be selected at the time of analysis. This is because the acquired raw images are distributed over all phases of the heart cycle, and the choice of a different synchronization phase simply causes different frames to be selected from the raw data. This makes retrospective gating particularly well-suited to 4D (3D + time) imaging, since the quantity of raw data required is independent of the number of different time points (heart phases) desired in the reconstructed movie, and the same raw data required for a single 3D reconstruction can just as easily be used to generate a 4D movie. However, the number of raw images acquired will still typically need to be several times more than the number actually selected for use in the final 4D reconstruction, and the data for each individual *z* slice will need to be acquired over the course of several successive heartbeats.

A guideline for the time required for post-acquisition analysis is 2–3 min for a stack of 20 slices each consisting of a time series of 225 images, 256 × 256 pixels (Liebling, 2014, Private communication). However, more sophisticated algorithms taking account of heart rate variations might be expected to take longer. Despite these demands on the data analysis, the original data acquisition is extremely simple, requiring only a video camera suitable for imaging at appropriate framerates. This makes it an attractive technique for biological researchers, since there is no need to invest in substantial local expertise and equipment in order to benefit from the technique.

### 3.2. Prospective gating

Consider now *prospective gating*, where the aim is to trigger image acquisition such that images are only acquired at the one desired phase in the heart cycle. Using our earlier example, the *only* frames acquired would be at 0.1 s, 0.6 s, 1.1 s, etc. In order to achieve this, some alternative form of input data is acquired in real time and is analyzed in order to determine the instantaneous phase of the heartbeat. This data is *not* generally the same imaging modality as that used for imaging the individual slices—indeed the use of a prospective approach may be motivated in part by a desire to reduce the exposure of the sample to fluorescence excitation light for the main imaging channel (Taylor et al., 2011). Alternatively, a number of authors have used a complimentary gating source that is more easily analyzed to calculate the heart phase. For example, Jenkins et al. (2007) used laser doppler velocimetry data from a non-invasive needle probe. Brau et al. (2002) and Sablong et al. (2014) used an independent optical fiber-based gating source, where a pair of fiber-optic cables were used to illuminate a region of tissue (esophagus or thorax, respectively) and collect the scattered light. Due to tissue motion the intensity of this scattered light is modulated in synchrony with the heartbeat.

In the case of a gating source consisting of a one-dimensional data channel (e.g., a scattered light signal measured on a photodiode, or a single velocity obtained from laser doppler velocimetry), peak detection algorithms can be used to identify a specific phase in the heart cycle. The principle here is exactly the same as ECG gating—and indeed commercial R wave detectors may be used for signal analysis. Alternatively, lock-in frequency analysis may be used if there is minimal frequency drift in the heart rate. These types of analysis can be implemented directly in electronic hardware.

**Figure 4:**
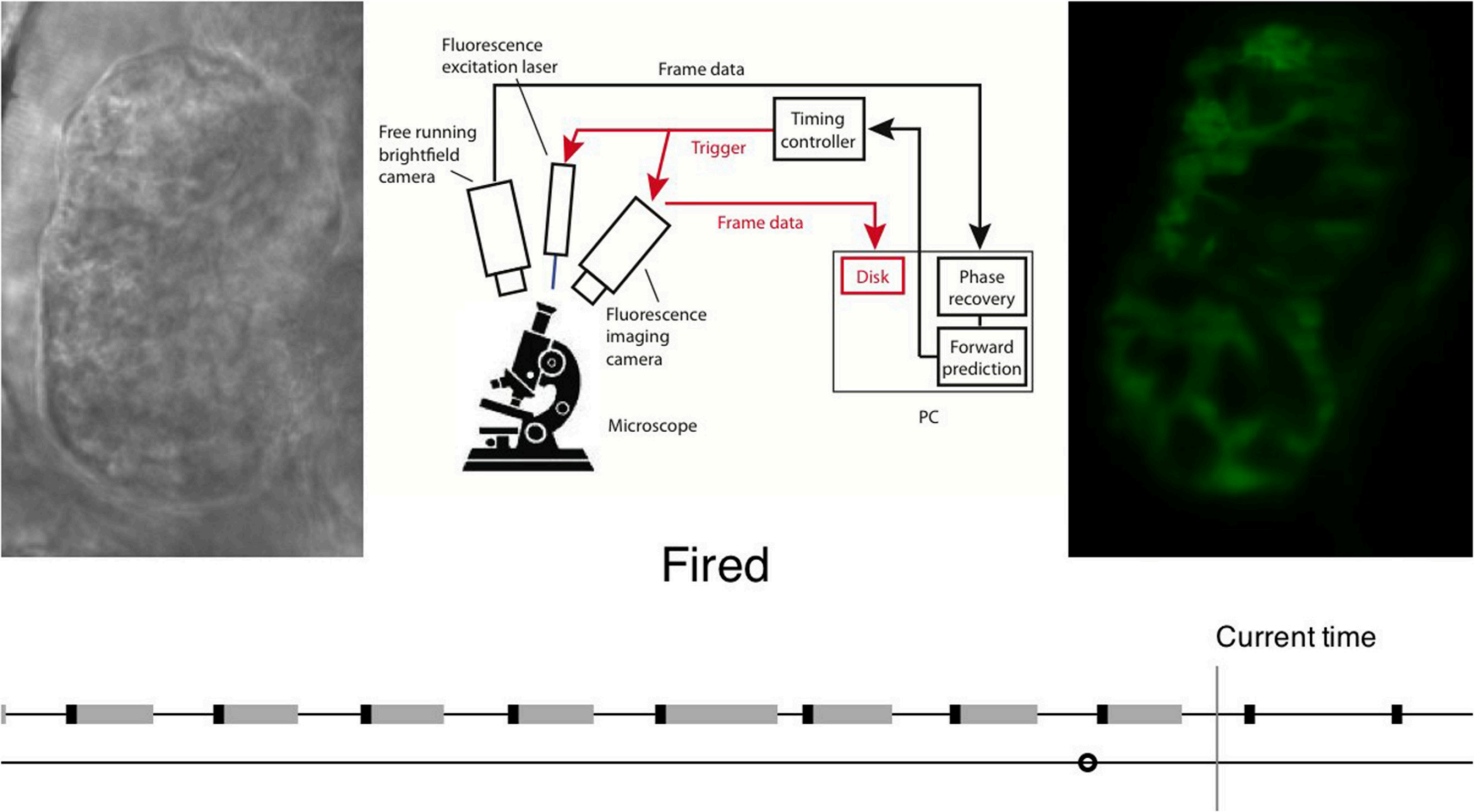
**Flow chart illustrating realtime image analysis for prospective optical gating**. The analysis process is explained in video form at http://www.opticsinfobase.org/boe/viewmedia.cfm?uri=boe-3-12-3043&seq=2). Image reproduced from Taylor et al. (2012) with permission.

If, however, video data is used as the gating source then analysis is more complex. Taylor et al. compared each received frame to one complete heartbeat of “reference frames,” and calculated a phase for each one based on the reference frame that it most closely resembled. In this case the processing time (of the order of 10 ms) and the relatively low sampling rate (video camera framerate 80 fps) means that it is no longer sufficient for the system to operate in a “reactive” mode where a trigger signal is sent as soon as the desired phase is observed. It is instead necessary for the design to acknowledge this latency: the system must extrapolate forwards in time in order to *predict* when the desired phase will be reached, in order for a trigger to be generated at the correct time and with sufficient temporal precision (Figure 4). This can then be used to trigger frame acquisition, and optionally also trigger simultaneous stroboscopic illumination in order to minimize exposure of the sample to light.

The temporal accuracy of a prospective gating system is not trivial to calculate from system parameters such as the sampling rate of the input data. However, two important factors affecting it are *accuracy of phase determination* and *latency* for signal generation (which implicitly affects the accuracy with which the correct trigger time can be *predicted*):

#### 3.2.1. Accuracy of phase determination

In the case of a single data channel, this depends on the accuracy with which a peak can be identified in the gating data, and this in turn depends on the signal-to-noise ratio of the input data. However, only a small number of such features in the waveform can be reliably and unambiguously identified in real time. Phases can be assigned to those few specific points, but in general it is not possible to identify the phase for an arbitrary point in the heartbeat. On the other hand in image-based prospective gating the phase can be determined to a similar degree of accuracy at all points in the cycle. For the algorithm used in Taylor et al. (2011) that accuracy will be a function of the framerate used in acquiring the reference frames, as well as potentially the quality of the images used for the gating analysis.

#### 3.2.2. Latency and prediction

Depending on the phase chosen for gated imaging, an appropriate delay may be required before the trigger signal is sent. In the case of single-channel gating, a trigger point could be selected immediately following the known phase point. However, even then there may be minor latency present: a peak can only be identified once the signal has been observed to decay again sufficiently that it can be identified as a true peak rather than an artifact due to random noise. More generally, if triggering is required at any other point in the cycle then the gating system must predict the time at which the heart will be at the desired phase point for imaging. This prediction may be implicit, in the form of a fixed time delay. However, the heart does not beat in a perfectly periodic manner, and variability between and within beats will mean that a higher latency will inevitably result in an increased temporal error between the correct time that the trigger should be sent and the time that it is actually sent (this variability is discussed by a number of authors including Lee et al., 2012; Taylor et al., 2012; Bhat et al., 2013). For this reason, Taylor et al. used recently-obtained phase/time information to make a forward prediction in time by a small amount (around 20 ms) in order to allow for the latencies due to frame acquisition (up to 12 ms at 80 fps), image processing (~ 5 ms) and serial communication (~2 ms).

In summary, the trigger accuracy of a prospective gating system is affected by the latency between when the last known phase point was determined and when the trigger pulse is generated, but it is important to emphasize that the temporal accuracy of the trigger pulse itself is *not* the same as the latency, and the former would normally be expected to be much smaller than the latter.

The computational demands of prospective gating depend on the algorithm and gating source used. They can be trivial (in the case of an electronic peak detection device), but analysis of video images as performed by Taylor et al. requires careful optimization of computer code in order that the necessary calculations can be completed in real time. Furthermore, prospective gating requires additional timing hardware to be interfaced with the imaging system for triggering (which may not always be possible), along with the necessary hardware for the gating source (e.g., optical fiber, brightfield camera port).

### 3.3. Inter-slice registration

An important consideration in 3D imaging is correct registration between image slices, regardless of the gating approach used. In other words, it is important that the phase computationally assigned to a snapshot at one depth in the heart matches that of the counterpart snapshot at a different depth in the heart. In early demonstrations of retrospective synchronization, Liebling et al. (2005) applied their algorithm to temporally align adjacent slices, but this was open to the accumulation of systematic errors over the full volume of the dataset. This can be avoided by designing a gating scheme that contains at least some information that is invariant over the course of the scan. That information can then be compared meaningfully *between* different slices and used to synchronize each slice relative to the others.

**Figure 5:**
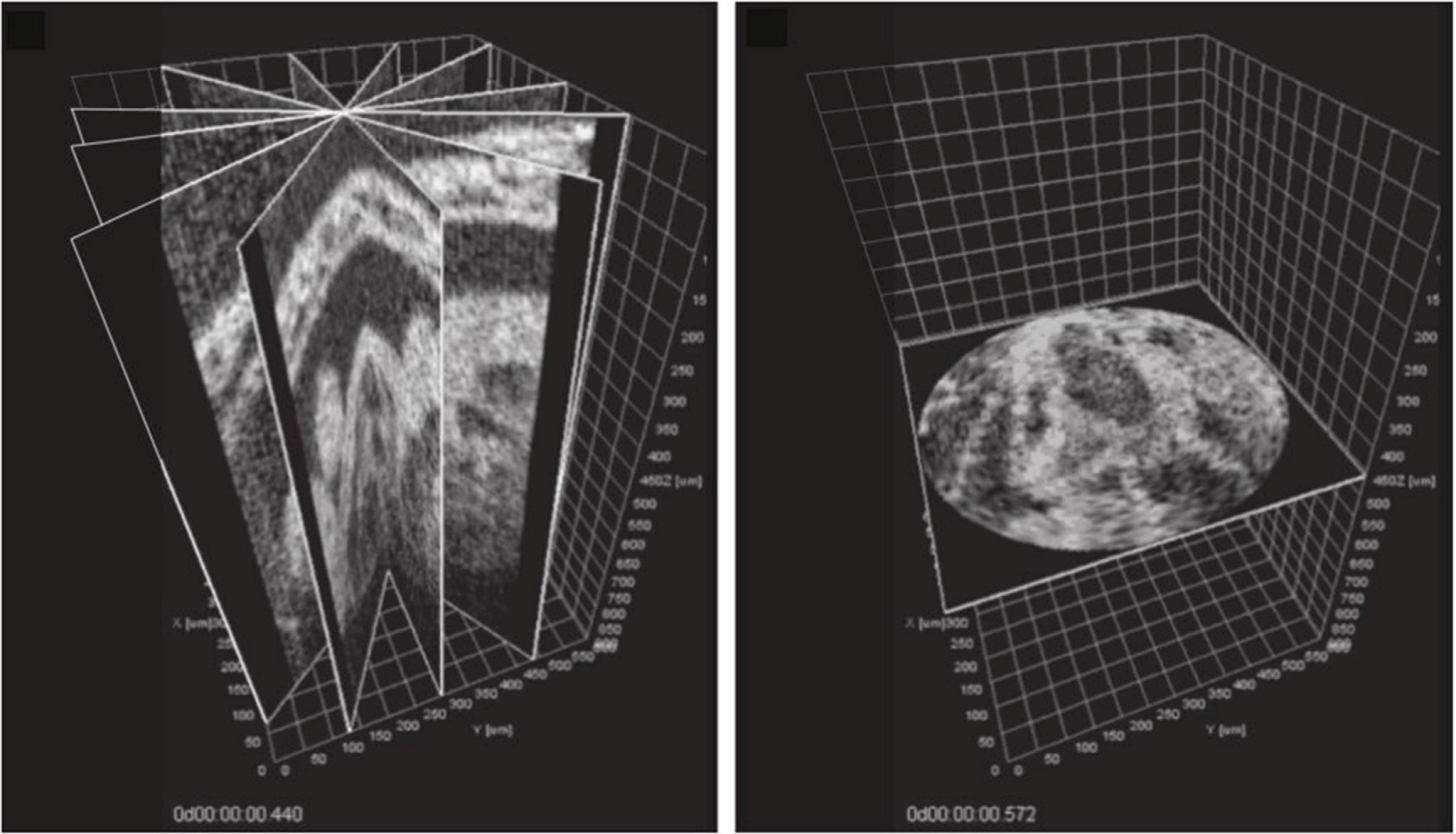
**Post-acquisition synchronization of a series of OCT slices acquired at different angles around a common axis (left) allows a temporal snapshot to be reconstructed in a perpendicular plane (right)**. See video at http://www.opticsinfobase.org/viewmedia.cfm?uri=boe-3-3-650-2. Images reproduced from Larina et al. (2012) with permission.

Happel et al. (2011) and Larina et al. (2012) used a rotational geometry to acquire 2D image slices, in which successive slices were obtained by rotation of the sample to different orientations, rather than performing a linear scan through the sample. The result of this is that all images share a central line (the axis of rotation) that images the same tissue regardless of the rotation of the sample. It is then possible to directly compare and align phases between different image slices, by analysis of that line of each image (Figure 5). This approach can be considered analogous to approaches used in gated MRI, where for example (Buehrer et al., 2008) describe interleaving repeated measurements of the *k*-space center in between slice acquisitions in order to obtain a slice-independent gating signal. A somewhat similar approach was taken by Liu et al. (2009), who acquired an OCT scan series that included a single reference scan image perpendicular to the main slice direction. The perpendicular scan intersected every slice scan, allowing each slice in turn to be synchronized to the reference scan even though there was no one single axis common to every image.

An alternative solution is to use an independent imaging path for the synchronization reference. In Taylor et al. (2012) an independent brightfield image channel was used for the synchronization analysis. Since they used the SPIM imaging modality (Selective [or Single] Plane Illumination Microscopy), with the sample being moved in order to achieve a *z* scan, a specific optical design was required to ensure that the brightfield images remained *z*-invariant, thus again allowing direct comparison of phases between different image slices. We note that techniques have been reported for fast volume scanning of a sample in confocal imaging (Botcherby et al., 2007) and SPIM imaging (Fahrbach et al., 2013; Mickoleit et al., 2014) that do not involve relative motion between sample and objective, and hence would not require this form of correction on the brightfield imaging path.

Finally, approaches using a completely independent gating source such as fiber-based light scattering measurements (Brau et al., 2002; Sablong et al., 2014) are inherently unaffected by *z* scanning of the imaging channel, meaning that inter-slice registration is ensured without any additional effort beyond that required to synchronize a single slice.

All these various approaches offer promising solutions to the problem of inter-slice registration, by ensuring that the information channel used for synchronization remains invariant under scanning.

## 4. Discussion

### 4.1. Non-invasive imaging

An additional consideration in the case of fluorescence imaging is the exposure of the specimen to laser light. This can lead to deleterious photobleaching and phototoxic effects, compromising imaging and potentially even affecting the normal development of an embryo (Lichtman and Conchello, 2005; Editorial, 2013), particularly at the accumulated doses associated with longer-term imaging. In a study of zebrafish hair cells (which did not require motion gating) (Swoger et al., 2011) noted that they had restricted their imaging interval to 200 ms every 4 min in order to be able to maintain imaging over a period of 12–18 h without suffering the effects of photobleaching—in spite of their use of the SPIM imaging modality, which involves substantially reduced light exposure compared to e.g., spinning-disk confocal imaging (Vermot et al., 2008, Table III). The issue of bleaching is a potential issue for retrospective gating of heart images, where typically many more images are acquired than are necessary for reconstruction of a 3D or 4D dataset, and this may limit the applicability of the approach or require specific allowances to be made for bleaching in the analysis of the images (Liebling et al., 2005). This issue is substantially reduced in the case of prospective gating, where no superfluous fluorescence images need to be acquired and the exposure of the sample can be limited to the equivalent total light energy that would be required in a non-moving sample—although this must be considered in combination with other strengths and weakness of the two approaches (see Section 3).

### 4.2. Computationally synthesized images

In much of our discussion we have referred to synchronization of 2D image slices in order to synthesize a 3D (or 4D) image. However, a number of advanced optical imaging techniques require more than one raw 2D data acquisition in order to computationally synthesize even a single 2D image—for example structured illumination, noise reduction and image optimization adaptive optics. For *in vivo* imaging affected by heart-related motion artifacts, such techniques can only be implemented if all the raw data acquisitions represent the same underlying scene—in other words they must represent the same heart phase. Since commonly-used optical synchronization algorithms do not generally make assumptions about the shape or form of the raw images (Liebling et al., 2005; Taylor et al., 2011) and/or analyze a separate imaging channel (Taylor et al., 2011), they are equally suitable for application to these computationally synthesized imaging techniques, extending their utility to the beating heart and other moving tissue.

Bhat et al. (2009) computationally combined OCT images from multiple heartbeats in a mouse embryo, averaging equivalent pixels from multiple cycles in order to synthesize an image with improved signal-to-noise ratio. Ohn et al. (2012) showed that it is possible to co-register datasets consisting of several independent fluorescence channels acquired one after the other, as long as there is sufficient co-localization of features between the channels to allow a statistical correlation to be identified between the sequences. Finally, Bourgenot et al. (2013) used prospectively gated images as input into an image-optimization adaptive optics scheme. Since in this last case the correction for each image is only determined following analysis of previously-acquired data, prospective gating is the only solution fast enough to be practical in this context.

### 4.3. Gated intervention

Prospective gating also opens up the possibility of gated intervention. For example, an ablation laser can be triggered in order to target a specific localized region of cells within the heart (Matrone et al., 2013). Without synchronization the accuracy that could be achieved would be drastically reduced, since limited human reaction times effectively mean that a manually-fired laser would be activated at a random point in the heart cycle. The ability to target highly localized regions of tissue enables experiments to be performed in the normally-beating heart where previously it would be necessary to pharmacologically stop and restart the heart. Thus, the non-invasive principle of optical gating can be extended from imaging to intervention and manipulation of beating heart tissue. Similar lower-power optical interventions could also offer the potential for photo-uncaging and optogenetic interaction with the normally-beating heart.

## 5. Conclusion

Optical gating has been demonstrated in a wide range of different imaging modalities, and in a number of different species. Table 1 lists some representative publications to illustrate this variety. The vast majority of publications refer to imaging of embryos; we speculate that this is largely due to a dearth of optical imaging techniques suitable for deep tissue imaging in most adult organisms, be that in the heart or in other organs. There is however no inherent reason why optical gating is not suitable for imaging of adult hearts, if appropriate imaging techniques are available.

**Table 1:** **Examples of the range of imaging modalities and species in which optically gated imaging has been demonstrated**.

**Reference**	**Modality**	**Gating type**	**Species**
Gargesha et al., 2009	OCT	Retrospective	quail
Jenkins et al., 2007	OCT	Prospective (LDV)	quail
Jenkins et al., 2012	OCT	Retrospective	quail
Mariampillai et al., 2007	OCT	Retrospective	*Xenopus laevis*
Bhat et al., 2013	OCT	Retrospective	rat, mouse
Peterson et al., 2012	Doppler OCT	Retrospective	quail
Taylor et al., 2012	SPIM	Prospective (brightfield)	zebrafish
Mahou et al., 2014	two-photon SPIM	Retrospective	zebrafish
Jamison et al., 2012	Brightfield/PIV	Retrospective	zebrafish
Ohn et al., 2009	Brightfield/PIV	Retrospective	zebrafish
Liebling et al., 2006a	Confocal line	Retrospective	zebrafish
Staudt et al., 2014	Spinning-disc confocal	Retrospective	zebrafish
Mattison et al., 2013	PA	Retrospective	zebrafish
Sablong et al., 2014	MRI	Prospective (fiber)	mouse

*(OCT, optical coherence tomography; LDV, laser doppler velocimetry; SPIM, selective plane illumination microscopy; PIV, particle imaging velocimetry; PA, photoacoustic microscopy; MRI, magnetic resonance imaging)*.

The main strengths of retrospective gating are the ease of acquisition of raw data, and the flexibility in choice of synchronization phase. Acquisition of raw data simply requires high-speed video data to be recorded in the chosen imaging modality. The large volume of data acquired can then be analyzed at a later time and location, for any desired heart phase.

In contrast, the main strengths of prospective gating are the reduced exposure of the sample to laser light (in the case of fluorescence imaging modalities) and the ability to perform synchronized intervention, as well as techniques such as adaptive optics that require a near-real-time response to the acquired image data. Because electrical signals are generated in real time to trigger image acquisition, no superfluous images are acquired, thus minimizing photobleaching and phototoxic effects in the sample.

The use of optical gating in beating-heart imaging has already enabled the discovery of significant biological findings about the development of the heart, such as work by Forouhar et al. (2006), who used 4D images of the embryonic zebrafish heart and blood cells to obtain evidence that the embryonic heart functions as a dynamic suction pump as opposed to a peristaltic pump, and (Liebling et al., 2006a) who used similar video sequences to investigate the transition of the heart's function from a suction pump to unidirectional flow mediated by a functioning atrioventricular valve. Garita et al. (2011) used 4D images of the embryonic quail heart to study the interactions between the endocardial and myocardial compartments over the course of the heartbeat. Gu et al. (2012) used 4D imaging in the course of their studies into the effects of hypoxic stress on the function and development of the quail heart, while (Staudt et al., 2014) used 3D time-lapse imaging over the course of around 12 h to study the processes involved in the formation of trabeculae within the ventricle of the zebrafish heart. In particular, they used optical gating to “freeze” the periodic motion of the heartbeat in order to be able to clearly see the slower-timescale motion of cell growth and migration within the tissue. Lin et al. (2014) have also shown the potential uses for 3D imaging in the context of drug screening. Finally, Goetz et al. (2014) used 4D imaging in support of their investigation into the role that endothelial cilia play in the sensing of shear forces in the developing cardiovascular system.

Optical gating allows 3D images and 3D + time (4D) movies to be obtained of the heart as it beats, free from motion artifacts, as well as offering the possibility of synchronized intervention. With optical gating now demonstrated in a range of different modalities and model animals, non-invasive high temporal and spatial resolution imaging is possible for the first time in living, moving cardiac tissue. This has opened up an as-yet-uncharted landscape of novel developmental and functional studies in the normally-beating heart, studying myocytes free from any external perturbation.

### Conflict of interest statement

The author declares that the research was conducted in the absence of any commercial or financial relationships that could be construed as a potential conflict of interest.
